# Synergistic Interface Energy Band Alignment Optimization and Defect Passivation toward Efficient and Simple‐Structured Perovskite Solar Cell

**DOI:** 10.1002/advs.201902656

**Published:** 2020-01-29

**Authors:** Like Huang, Danli Zhang, Shixiao Bu, Ruixiang Peng, Qiang Wei, Ziyi Ge

**Affiliations:** ^1^ Ningbo Institute of Materials Technology and Engineering (NIMTE) Chinese Academy of Sciences (CAS) Ningbo 315201 China; ^2^ Center of Materials Science and Optoelectronics Engineering University of Chinese Academy of Sciences Beijing 100049 P. R. China

**Keywords:** electron transport layers, energy level alignment, nonconjugated small molecules, perovskite solar cells, work function

## Abstract

Efficient electron transport layer–free perovskite solar cells (ETL‐free PSCs) with cost‐effective and simplified design can greatly promote the large area flexible application of PSCs. However, the absence of ETL usually leads to the mismatched indium tin oxide (ITO)/perovskite interface energy levels, which limits charge transfer and collection, and results in severe energy loss and poor device performance. To address this, a polar nonconjugated small‐molecule modifier is introduced to lower the work function of ITO and optimize interface energy level alignment by virtue of an inherent dipole, as verified by photoemission spectroscopy and Kelvin probe force microscopy measurements. The resultant barrier‐free ITO/perovskite contact favors efficient charge transfer and suppresses nonradiative recombination, endowing the device with enhanced open circuit voltage, short circuit current density, and fill factor, simultaneously. Accordingly, power conversion efficiency increases greatly from 12.81% to a record breaking 20.55%, comparable to state‐of‐the‐art PSCs with a sophisticated ETL. Also, the stability is enhanced with decreased hysteresis effect due to interface defect passivation and inhibited interface charge accumulation. This work facilitates the further development of highly efficient, flexible, and recyclable ETL‐free PSCs with simplified design and low cost by interface electronic structure engineering through facile electrode modification.

## Graphical abstract

A polar nonconjugated small molecule ultrathin layer with an intrinsic dipole moment is introduced to modify the work function of indium tin oxide and to optimize the front interface energy level alignment, which contributes to suppressed energy loss and results in a 20.55% efficient electron transport layer–free perovskite solar cell with enhanced open‐circuit voltage short circuit current density and fill factor, simultaneously.

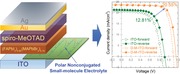

## 1 Introduction

In the last decade, perovskite solar cells (PSCs) have attracted great attention with their unparalleled increase of power conversion efficiency (PCE) from the initial 3.8%^1^ to the current record of 25.2%^2^ by virtue of intensive materials and interface engineering.^3^, ^4^ The excellent ambipolar charge transport properties of perovskite family^5^ as well as the complex and energy intensive electron transport layer (ETL)^6^ and its associated UV‐light instability of the whole device^7^ soon lead to the invention of ETL‐free PSCs^8^, ^9^, ^10^ with much simplified structure, reduced processing steps, and lower production cost, hence great potential in large‐area flexible application.^11^ Many pioneering works have been reported on ETL‐free PSCs.^8^, ^11^, ^12^ However, all present ETL‐free PSCs, unfortunately, suffer from low PCEs compared to that of the hole transport layer (HTL)‐free PSCs with a highest PCE of 20.2%.^13^

Generally, the absence of an ETL in the device can cause unfavorable band bending and energy level alignment with a large energy barrier for electrons (Δ*E*_e_) to transport across the transparent conductive oxide (TCO) electrode/perovskite interface and a small energy barrier for holes (Δ*E*_h_) blocking at this interface due to mismatched energy level, which fundamentally limits the performance of ETL‐free PSCs. Here, Δ*E*_e_ is defined as the energy difference between the Fermi level (*E*_F_) of the TCO electrode and the conduction band maximum (CBM) of perovskite.^14^ While, Δ*E*_h_ is the energy difference between the *E*_F_ of the TCO electrode and the valence band maximum of perovskite. Δ*E*_e_ and Δ*E*_h_, in fact, correspond to the free energy change of charge carrier when they transport across the TCO/perovskite interface. For efficient ETL‐free PSCs, it is urgently desired to reduce or wholly eliminate such an electron barrier. To achieve this, the work function (WF) of TCO and/or perovskite should be carefully regulated individually or simultaneously to flip the direction of the interface band bending and to decrease Δ*E*_e_. In theory, the former requires that the WF of TCO is smaller than that of perovskite, i.e., WF_TCO_ < WF_perovskite_, and the latter needs that the CBM of perovskite should be much closer to the *E*_F_ of TCO. In fact, previous work has suggested that the WF of cathode has a great influence on the performance of planar device.^15^

As for the energy level optimization of perovskite at one side of the TCO/perovskite interface, in our previous work,^10^ by intentional polarity tailoring of MAPbI_3−_ *_x_*Cl*_x_* perovskite via finely control the concentration of incorporated n‐type dopant, ETL‐free PSCs with optimized energy level alignment both at fluorine‐doped tin oxide (FTO)/MAPbI_3–_ *_x_*Cl*_x_* and MAPbI_3–_ *_x_*Cl*_x_*/spiro‐MeOTAD interfaces are constructed, resulting in enhanced built‐in electric field thus efficient majority carrier collection and minority blocking as well as reduced interfacial charge recombination. The resultant ETL‐free device with such a difficult to achieve Schottky/p‐n cascade heterojunction, however, only achieved a maximum PCE of 12.62%. Obviously, engineering the CBM and WF of perovskites to minimize Δ*E*_e_ at the TCO/perovskite interface is quite complex and space limited, since the electronic structure of perovskites depends sensitively on the specifics compositions and deposition conditions. Hence, the space of the device performance improvement through the energy level optimization of perovskite is also limited.

Another strategy to efficiently minimize Δ*E*_e_ is to shift the *E*_F_ of TCO at another side of the TCO/perovskite interface toward the CBM of perovskite. Although the WF of commercial TCO is given (4.70 eV for ITO and 4.40 eV for FTO) and their *E*_F_ is difficult to change by post‐doping of the bulk, the WF of TCOs can still be decreased considerably by pulling down the surface vacuum level (*E*_sur‐vac_) via simple solution coating of ultra‐thin polar organic modifying layer (ML) as widely used in organic electronics.^16^ Despite this, organic (especially nonconjugated) ML has never been utilized to fabricate high‐efficiency ETL‐free PSCs.

In this work, a room temperature spin‐coated polar nonconjugated small‐molecule electrolyte (PNSME) layer, namely 4,4′‐(((methyl(4‐sulphonatobutyl)ammonio)bis(propane‐3,1‐diyl))bis(dimethyl ammoniumdiyl))bis‐(butane‐1‐sulfonate) (MSAPBS, Figure S1, Supporting Information),^17^ is adopted as an organic ML in ETL‐free PSCs to effectively enhance the device performance. It is found that the MSAPBS ML can chemically bind to the ITO surface by forming strong S—Sn and S—In bonds, as identified by X‐ray photoelectron spectroscopy (XPS) analysis. With an intrinsic dipole moment, the PNSME ML can significantly decreases the *E*_sur‐vac_, thus the WF of ITO decreases greatly from 4.70 to 3.73 eV (and further to 4.00 eV with *N*,*N*′‐dimethylformamide (DMF) washing). The decreased WF of ITO reverses the band‐bending direction of the perovskite from upward to downward, which dramatically decreases the Δ*E*_e_ from 0.65 to −0.05 eV, while the Δ*E*_h_ is increased from 0.93 to 1.63 eV. Thereby, the electron extraction efficiency and hole blocking efficiency at the ITO/perovskite interface are greatly improved, while the charge recombination and energy loss are effectively decreased, giving rise to a greatly enhanced open‐circuit voltage (*V*_oc_), short circuit current density (*J*_sc_), and fill factor (FF), simultaneously. Accordingly, the PCE of the modified device reaches 20.55%, comparable to state‐of‐the‐art PSCs with an ETL, while PCE of the PSCs on bare ITO is only 12.81%.

## 2 Result and Discussion

**Figure 1:**
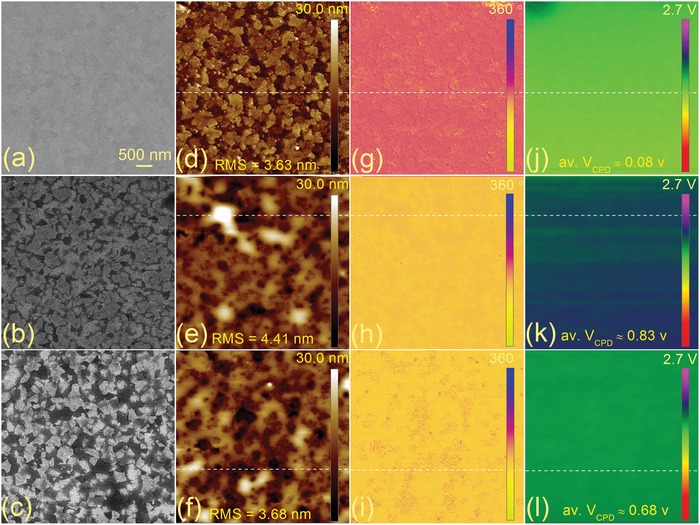
Surface SEM images of a) pristine ITO, b) M‐ITO, and c) D‐M‐ITO. d–f), g–i), and j–l) present their corresponding topographic AFM images, phase‐contrast AFM images, and CPD derived from KPFM measurement. The area of all the images is 5 µm × 5 µm.

MSAPBS is easily soluble in polar solvents, including water, methanol, and dimethyl sulfoxide (DMSO), but is insoluble in the nonpolar solvents, such as chlorobenzene, dichlorobenzene, and chloroform, which is favorable for solution‐processed PSCs.^17^ The MSAPBS ML is deposited on ITO at room temperature by simple spin‐coating of methanol solution without any further thermal annealing treatment. **Figure** **1** shows the surface topography and potential change of ITO before and after MSAPBS modification (M‐ITO) as derived from scanning electron microscopy (SEM) and Kelvin probe force microscopy (KPFM) measurement. Considering the fact that the strong polar solvent DMF of perovskite precursor may dissolve part of the MSAPBS film during spin‐coating of perovskite precursor solution, the measurement results of M‐ITO washed with DMF (D‐M‐ITO) with the same volume of the perovskite film precursor solution are also given (energy‐dispersive X‐ray spectroscopy measurement in Figure S2, Supporting Information). In order to avoid severe electron beam damage of the MSAPBS film, SEM is performed using ultra‐low voltage of 2 kV. From Figure 1a–c, MSAPBS modification makes the surface SEM of ITO look black, which is often encountered when organic films are deposited on the substrate surface. While, further DMF washing makes it slightly brighter, possibly due to partial removal of MSAPBS by DMF. The root mean square (RMS) of the three films derived from topographic atomic force microscopy (AFM) images (Figure 1d–f) is 3.63, 4.41, and 3.68 nm, respectively. This indicates that the MSAPBS modification slightly improves the surface roughness of ITO, while the DMF rinse reduces the roughness of the M‐ITO surface to the value close to pristine ITO. This is further demonstrated by the sectional morphology of the three films as shown in Figure S3a in the Supporting Information. To confirm that DMF washing does not seriously dissolve and completely remove MSAPBS, the phase‐contrast AFM images of the three films are also given. In general, the difference in phase implies a variation in elastic modulus and viscosity coefficient of the sample surface.^18^ From Figure 1g–i, the phase‐contrast AFM images of M‐ITO and D‐M‐ITO are similar (with little difference in the pit area of D‐M‐ITO), while they are significantly different from that of the pristine ITO, which suggests that DMF washing does not remove all the MSAPBS film completely. This is quantitatively supported by the average phase angle of the three films as shown in Figure S3b in the Supporting Information. As a contact‐less, nondestructive, and easy‐performable method, KPFM can directly measure the contact potential difference (CPD), between a metal tip and the sample surface, which corresponds to the difference in their WFs.[qv: 19a] Thus, KPFM was then employed to investigate the ITO surface potential change due to WF decrease induced by MSAPBS modification. The relative values in WF of ITO and modified ITOs can be determined by the CPD

(1) $$V_{\text{CPD}}\;\;=\;\;\frac{\varphi_{\text{tip}}-\varphi_{\text{sample}}}{e}$$

where φ_sample_ and φ_tip_ are the WFs of the sample and tips, and *e* is the element charge. Using the same scanning tip during the measurement, φ_tip_ can be treated as a constant value. Figure 1j–l presents the spatial potential maps of ITO and modified ITOs. Figure S3c in the Supporting Information shows variations in local CPD across the three samples surface. In spite of the larger morphological and RMS change of modified ITOs as suggested by Figure 1d–f and the sectional height change (Figure S3b in the Supporting Information, even some pinholes of MSAPBS appear for D‐M‐ITO), the CPD of the modified ITOs is much more uniform (with a fluctuation amplitude of about 0.02 V for M‐ITO and D‐M‐ITO) than the pristine ITO (with a fluctuation amplitude of about 0.06 V). Figure S4 in the Supporting Information further suggests that the MSAPBS film coverage with the presently discussed thickness has little effect on the homogeneity of M‐ITO work function. Generally, the uniformity of the surface work function of the carrier collecting electrode directly determines the uniformity of its carrier collection,[qv: 19b] although the specific effect of the microscopic uniformity of carrier collection on the macroscopic devices efficiency plane distribution is unknown presently. In the present work, MSAPBS layer is deposited by spin‐coating method. With this technique, always, there is uniformity issues. Interestingly, in spite of the pinholes in the modification layer the work function distribution of the resulted ITO is still very uniform, indicating great potential in large area deposition and application. Presently, we think this can be explained by the electrostatic theory of metals under equilibrium:^20^ a conductor under electrostatic equilibrium is an equipotential body with equal potentials everywhere and its surface is an equipotential surface. Similar results have also been reported in previous works where even discontinuous Cs_2_CO_3_ sub‐microscale crystal spots on the ITO surface can tune its surface WF homogenously and efficiently.^21^, ^22^ Moreover, we infer that the main factor affecting the surface WF value of modified ITO is the thickness of the modified layer, while the pinholes in the modified layers with different thickness only cause a small fluctuation of the surface WF value distribution of the modified ITO as shown in Figures S3 and S4 in the Supporting Information. More detailed research will be carried out in another work.

**Figure 2:**
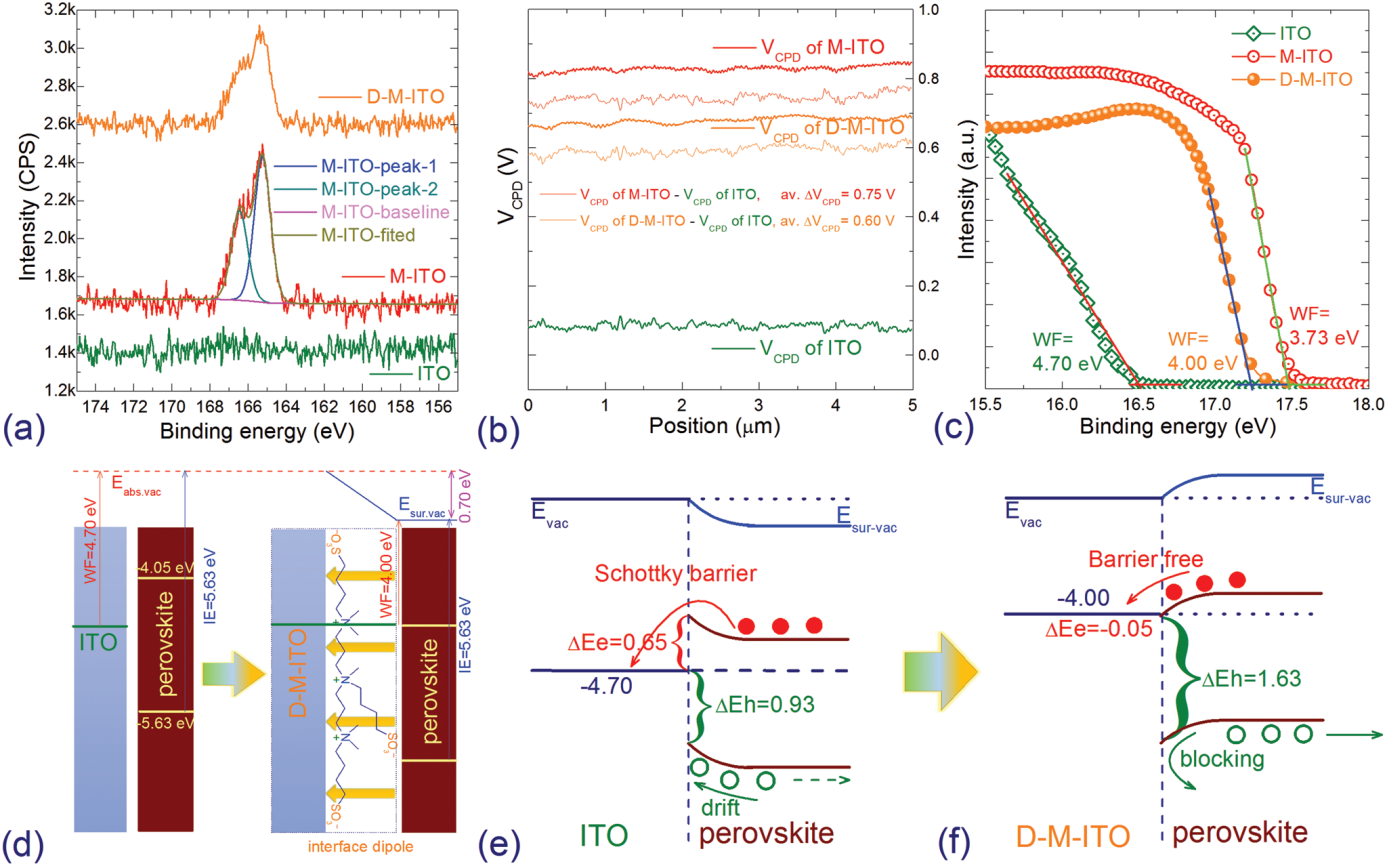
a) XPS spectra of the S 2p core levels, b) CPD and c) UPS spectra of the secondary electron cutoff region of ITO and D‐M‐ITO. d) Schematic illustration of the energy level diagram of the ITO/perovskite interface before and after modification. Interface energy levels alignment of e) ITO/perovskite and f) D‐M‐ITO/perovskite. Unit of all energy levels: eV.

XPS was carried out to investigate the surface composition of the modified ITOs to confirm the existence of MSAPBS. As shown in **Figure** **2**a, for ITO no photoelectron emission peak corresponding to the elements of MSAPBS can be observed. For M‐ITO, two main peaks located at binding energy (BE) of 166.45 and 165.25 eV are assigned to 2p core levels of characteristic element S, respectively.^23^ Further DMF washing decreases the intensity of these two peaks slightly, suggesting that the MSAPBS film has not been cleaned out completely. The evolution of In 3d and Sn 3d core levels in Figure S5 in the Supporting Information also supports this speculation. From Figure 2b, the *V*_CPD_ of M‐ITO and D‐M‐ITO is higher than that of pristine ITO by 0.75 and 0.60 V, respectively. According to the definition of CPD, the WF difference between ITO and modified ITOs can be determined by their difference in *V*_CPD_

(2) $$\Delta V_{\text{CPD}}\;\;=\;\;V_{\text{CPD}\;\text{of}\;\text{Mod-ITO}}\;\;-V_{\text{CPD}\;\text{of}\;\text{ITO}}=\frac{\varphi_{\text{tip}}-\varphi_{\text{Mod-ITO}}}{e}-\frac{\varphi_{\text{tip}}-\varphi_{\text{ITO}}}{e}=\frac{\varphi_{\text{ITO}}-\varphi_{\text{Mod-ITO}}}{e}$$

This suggests that their WF is lower than ITO by 0.75 and 0.60 eV, respectively. This trend is almost identical to the results given by the ultraviolet photoelectron spectroscopy (UPS) measurements. From Figure 2c, the WF of ITO, M‐ITO, and D‐M‐ITO is 4.70, 3.73, and 4.00 eV, respectively. Obviously, MSAPBS modification reduces the WF of ITO by ≈0.97 eV; while further DMF washing during spin‐coating of perovskite film reduces the MSAPBS film thickness and leads to a slightly enhanced WF of M‐ITO (3.73 eV) to 4.00 eV of D‐M‐ITO. The fact that DMF treatment partially remove MSAPBS layer, thus the D‐M‐ITO surface becomes smoother than M‐ITO and the lower value of WF of the D‐M‐ITO than M‐ITO further suggests that the WF of the modified ITO depends on the thickness of the modified layer as mentioned above. The energy level alignment of ITO and perovskite before and after modification determined by UPS is shown in Figure 2d (UPS of perovskite is shown in Figure S10, Supporting Information). The dipoles at ITO/perovskite interfaces introduce an electrical field across the MSAPBS layer pointing from perovskite toward the ITO surface, which results in an *E*_sur‐vac_ down shift at the surface of ITO and finally leads to the reduction of the effective WF at the surface of ITO (Figure 2d). The effective work function (WF_eff_) of ITO is determined by^14^

(3) $${\text{WF}}_{\text{eff}}\;\;=\;\;\text{WF}+E_{\text{ID}}$$

where *E*_ID_ (‐0.7 eV) is the energy of the interface dipole and WF (=4.70 eV) is the work function of ITO. The large *E*_ID_ can be caused by the sum of all molecular dipole moments of the whole MSAPBS layer.

The schematic band diagrams of ITO or D‐M‐ITO and perovskite after contact determined by UPS are shown in Figure 2e,f. Due to the difference in WF of ITO and perovskite, Schottky barrier associated with spontaneous band bending can be formed when these two materials contact. For ITO/perovskite interface, a large Δ*E*_e_ of 0.65 eV and a small Δ*E*_h_ of 0.93 eV with upward band‐bending exists due to the mismatched energy levels and WF, which is not favorable for electron collection and hole blocking and could results in severe interface charge accumulation and carrier recombination loss (Figure 2e and Figure S11a,b, Supporting Information). Interface charge accumulation can also lead to reduced device stability and enhanced hysteresis. Even if the electrons are successfully transferred, the free energy loss is significant due to the remarkable Δ*E*_e_, which can greatly decrease the *V*_oc_. While, for D‐M‐ITO/perovskite interface with matched energy levels, Δ*E*_e_ is reduced to be as small as −0.05 eV and Δ*E*_h_ is enhanced to be 1.63 eV with favorable downward band‐bending, due to the lowed *E*_sur‐vac_ of D‐M‐ITO and perovskite with assistance of the interfacial dipole (Figure 2f). This downward band bending in the perovskite as a driving force not only facilitates efficient electrons transfer and extraction by ITO but also helps blocking holes transfer from perovskite to ITO, which favors reducing the interface‐mediated carrier recombination and is expected to contribute to improved device performance. Note that, the M‐ITO has a low WF of 3.73 eV and this can theoretically lead to a negative Δ*E*_e_ of −0.32 eV, which is not favorable for electron extraction by M‐ITO (Figure S11c,d, Supporting Information). In spite of this, fortunately, the unintentional DMF washing during perovskite deposition then slightly increases the WF of M‐ITO to 4.0 eV by decreasing the MSAPBS thickness, resulting in an almost negligible Δ*E*_e_ of −0.05 eV at the D‐M‐ITO/perovskite interface (Figure S11e,f, Supporting Information). Therefore, we emphasize that in the current situation, the unintentional post‐treatment of DMF also contributes to the above‐mentioned energy levels alignments and the observed device performance improvement after mention.

Figure S12 in the Supporting Information gives the energy levels of a) isolated (FAPbI_3_)_0.85_(MAPbBr_3_)_0.15_ perovskite and b) spiro‐MeOTAD HTL before contact. Obviously, perovskite and spiro‐MeOTAD show n and p‐type polarity here, respectively. According to the general theory of semiconductor heterojunction, the energy levels alignment of the p‐type spiro‐MeOTAD and n‐type (FAPbI_3_)_0.85_(MAPbBr_3_)_0.15_ perovskite after contact thus can be sketchily drawn (c). The p‐n heterojunction with favorable energy band bending and large Δ*E*_e_ (=1.80 eV), small Δ*E*_h_ (=0.41 eV) at this interface can ensure efficient electron blocking and hole extraction.

**Figure 3:**
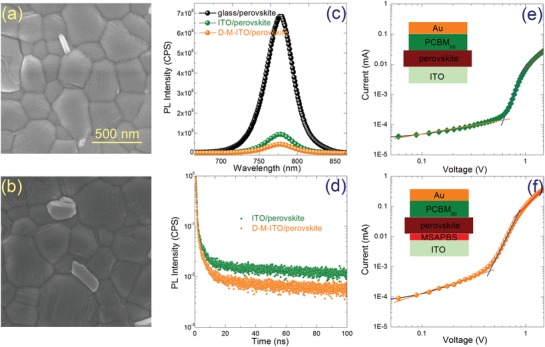
SEM images of perovskite films (1.5 µm × 1.5 µm) deposited on a) ITO and b) D‐M‐ITO. c) Steady‐state PL and d) TRPL spectra of the perovskite film on ITO and D‐M‐ITO. Dark *I*–*V* curves of the electron‐only devices with perovskite film on e) ITO and f) D‐M‐ITO.

Modification not only changes the surface electronic structure of ITO but also may alter the wettability of the substrates, which may further influence the nucleation and the surface morphology of the perovskite film deposited subsequently.^24^ Figure S6 in the Supporting Information gives the contact angle measurement of ITO, M‐ITO, and D‐M‐ITO. As can be seen, MSAPBS modification slightly decreases the surface wettability of ITO, while further DMF washing slightly increases the wettability of M‐ITO. In spite of these subtle changes, all substrates show good wettability that can ensure full surface coverage of perovskite film. **Figure** **3**a,b presents the SEM images of the perovskite films deposited on ITO and D‐M‐ITO, respectively. Figure S7 in the Supporting Information further gives the low‐magnification SEM and AFM images of the corresponding perovskite films, which reveals that the perovskite film deposited on D‐M‐ITO composes of larger grains. The increased grain size of the perovskite film deposited on D‐M‐ITO may be due to the slight decrease of ITO surface wettability, which reduces the nucleation density of perovskite precursors during nucleation (Figure S8, Supporting Information) as previous report.^24^ Figure S9 in the Supporting Information presents the a) XRD patterns and b) UV‐vis absorbance spectrum of the corresponding perovskite films. It suggests that the perovskite film deposited on D‐M‐ITO exhibits higher crystallinity and absorbance, which is well consistent with the results of SEM and AFM above. Figure 3c shows the steady‐state photoluminescence (PL) spectra of the perovskite films. Compared with the perovskite films deposited on glass and pristine ITO, larger extent of PL quench is observed in the D‐M‐ITO/perovskite, demonstrating the superior electron extraction capacity of D‐M‐ITO with much lower WF.^25^ Figure 3d shows the normalized time‐resolved PL (TRPL) for perovskite films, which demonstrates a much faster PL decay of the D‐M‐ITO/perovskite with stronger electron extraction capacity. The electron‐only devices ITO/(MSAPBS)/perovskite/PCBM/Au were fabricated to evaluate the trap density of perovskite films deposited on different substrates. Figure 3e,f shows the dark *I*–*V* curves of such devices. The linear correlation (orange) reveals an ohmic‐type response at low bias voltage, when the bias voltage is above the kink point, which defines as the trap‐filled limit voltage (*V*_TFL_), the current nonlinearly increases (blue), indicating that the traps are completely filled. The trap density (*N*_t_) can be obtained by^25^

(4) $$N_{\text{t}}\;\;=\;\;\frac{2\varepsilon_{0}\varepsilon V_{\text{TFT}}}{eL^{2}}$$

where ε_0_ is the vacuum permittivity, ε is the relative dielectric constant of (FAPbI_3_)_0.85_(MAPbBr_3_)_0.15_ (≈47),^26^ *e* is the electron charge, and *L* is the thickness of the film. The trap densities of the perovskite films coated on ITO and D‐M‐ITO substrates are 2.92 × 10^16^ and 2.07 × 10^16^ cm^−3^, respectively. The significantly decreased trap density is related to lower grain boundary density associated with slightly lager grain size and the passivation of interface defects due to the interaction between functional groups of MSAPBS and perovskite. As the S=O of the sulfonate group (SO^3−^) of MSAPBS can passivate the defect at the MSAPBS/perovskite interface as previously reported.^27^ Considering the solubility of MSAPBS in DMF, the modification may also passivate the grain boundary defects at the bottom of perovskite film. It is worth mentioning that passivation of front interface defects is also very important for improving device performance. Our previous work on device simulation of MAPbI_3_‐based ETL‐free PSC suggests that the quality of TCO/perovskite front interface as illuminated side has greater impact on the device parameters than that of the perovskite/HTL rear interface due to the large absorption coefficient of perovskite and the resulted exponential decay of absorption intensity thus photoexcited carrier concentration along the incident direction of light.[qv: 28a]

**Figure 4:**
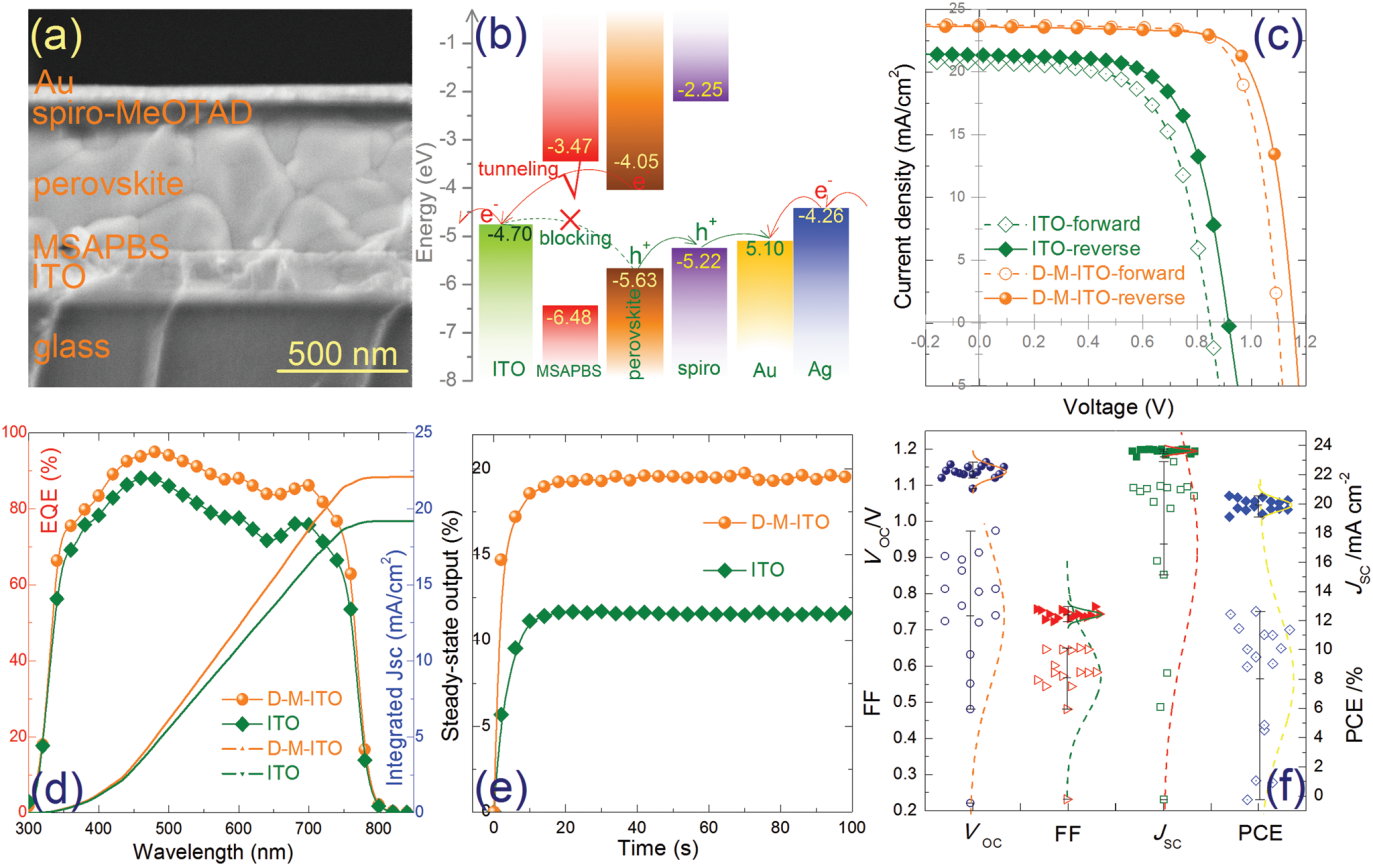
Device architecture and photovoltaic properties. a) Cross‐sectional SEM image and b) energy level diagram of ETL‐free PSCs. c) *J*–*V* curves of PSCs based on ITO and M‐ITO with different scanning directions. d) EQE spectrum and e) steady‐state output curve of the devices. f) Distribution of PCEs based on 30 individual devices for PSCs based on ITO and M‐ITO.

To further investigate the effect of the MSAPBS ML, ETL‐free PSCs are fabricated by using D‐M‐ITO and bare ITO as substrates, respectively. **Figure** **4**a,b shows the cross‐sectional SEM image and corresponding energy‐level diagram of the devices based on D‐M‐ITO, respectively. With a wide band gap, a nanometer‐thick MSAPBS film exhibits very weak absorption in UV‐vis wavelength. Thus, MSAPBS modification does not cause significant parasitic light absorption loss of the perovskite absorber (Figure S13, Supporting Information). Figure S14 in the Supporting Information presents the UPS spectra of ≈8 nm thick (optimized thickness for final device) MSAPBS film and its energy level position derived from the UPS measurement. Note that these values are different from that derived from cyclic voltammetry measurement in our previous work.^17^ In general, the energy levels of a single molecule are quite different from those of its condensed film. Especially for the case here, MSAPBS molecules have large dipole moment, which may lead to strong Coulomb electrostatic interaction between molecules via dipole interaction in the state of solid film. Although, with its intrinsic nonconjugated nature, MSAPBS itself is not an electronic conductor,^17^ electron can tunnel through the 8–10 nm insulated MSAPBS film and be collected by ITO electrode with the promotion of downward band bending at the D‐M‐ITO/perovskite interface as shown in Figure 2f. While, holes will be blocked by the electron insulated nature of MSAPBS layer and the large hole barrier at this interface. Therefore, MSAPBS is an effective interface modifier both from the optical and electronic perspectives. Figure 4c presents *J*–*V* curves of the champion device based on D‐M‐ITO and the reference device based on bare ITO. The ITO‐based device possesses a 12.81% PCE when being scanned in reverse. In sharp contrast, the champion device based on D‐M‐ITO presents a PCE as high as 20.55%, which is the highest PCE for ETL‐free PSCs reported so far. The improved performance of the D‐M‐ITO‐based devices is a comprehensive result from the enhanced *V*_oc_, *J*_sc_, and FF, simultaneously, as shown in **Table** **1**. Note that the *V*_oc_ of the ETL‐free PSC based on D‐M‐ITO reaches 1.15 V, which is comparable to that of the ETL‐based n‐i‐p PSCs with more complex design. Figure S15 in the Supporting Information presents the *J*–*V* curves (reverse scan) of ETL‐free PSCs with and without MSAPBS modification under a) AM1.5 solar illumination and b) dark. The enhanced threshold voltage in the dark curve is consistent with the enhanced *V*_oc_ of the device under illumination, confirming the improved rectifying property of the device with MSAPBS modification. To quantify the degree of hysteresis, we adopt a hysteresis factor (HF) defined in previous work (Equation (S1), Supporting Information).[qv: 28b] The HF for modified device is only 0.06, comparing to 0.14 for control device. Although the devices were not strictly hysteresis‐free, such hysteresis is much lower than that of a planar device (0.15, Figure S16, Supporting Information). This should be contributed to the reduced Δ*E*_e_ and enlarged Δ*E*_h_ that is favorable for charge extraction as well as the defect passivation effect as mentioned above. Device simulation was also conducted to further reveal the effect of ITO WF on the device performance (Figure S17, Supporting Information). Thus, our results also identify that rational engineering of the WF of electrode can be a possible avenue to mitigate the hysteresis issue. Figure 4d shows the external quantum efficiency (EQE) of the champion device. The integrated *J*_sc_ (=22.10 mA cm^−2^) from the EQE measurement confirms the accuracy of our *J*–*V* test. The enhanced EQE values in the whole UV‐vis wavelength range, especially in the short wavelength range, indicates the front interface defect passivation by functional SO^3−^ of MSAPBS as mentioned above. Figure 4e shows the steady‐state output of the two cells. It is observed that steady‐state output PCE increases from 11.50% to 19.72% after MSAPBS modification, which is consistent with the above *J*–*V* measurements. This observation is in good agreement with better matched energy level alignment based on D‐M‐ITO. To demonstrate the uniformity and reproducibility of the device, the statistical distribution of photovoltaic parameters based on 16 individual devices for PSCs based on D‐M‐ITO and ITO is shown in Figure 4f, which suggests an overall improvement of all device parameters of the PSCs based on D‐M‐ITO. We further tracked the stability of the devices under dark in glove‐box filled with N_2_ (oxygen and water content less than 0.1 ppm). Figure S18 in the Supporting Information shows that our D‐M‐ITO‐based device can keep ≈99% of the original PCE value after 60 days storage, while the ITO‐based device maintains 93% of its original PCE. The enhanced stability of the D‐M‐ITO‐based device can be due to the matched energy level alignment that ensures enhanced charge extraction and inhibited interface charge accumulation.^29^ Figure S19 in the Supporting Information demonstrates the universal applicability of this electrode design with more TCO electrode/perovskite combination. Due to its simple structure, the device can be readily recycled via a facile solution process at room temperature to further cut down the fabrication cost (Figures S20–S22, Supporting Information) and to avoid the possible lead pollution.^30^, ^31^

**Table 1:** Photovoltaic parameters of ETL‐free PSCs with and without MSAPBS modification

Device	Scan direction	*V*_oc_ [V]	*J*_sc_ [mA cm^−2^]	FF [%]	PCE [%]	Average PCE [%]	*R*_s_ [Ω]	*R*_sh_ [Ω]
ITO	Forward	0.85	20.71	62.80	11.05	11.93	7.00	40338.18
	Reverse	0.92	21.34	65.27	12.81		6.66	45599.54
ITO/MSAPBS	Forward	1.10	23.74	73.94	19.31	19.93	3.46	101521.58
	Reverse	1.15	23.62	75.67	20.55		3.13	122145.73

## 3 Conclusion

In conclusion, high‐efficient, hysteresis‐less, robust, and recyclable ETL‐free PSCs based on a thin polar nonconjugated small‐molecule electrolyte layer and the underlying mechanism have been demonstrated in this work. The MSAPBS ML with intrinsic molecular dipole moment forming an interfacial dipole at the ITO surface, which pulls down the surface vacuum level and effective WF of ITO by 0.97 eV, establishing better ohmic contacts between the (FAPbI_3_)_0.85_(MAPbBr_3_)_0.15_ perovskite and ITO. This electron‐selective contact with matched energy level alignment could facilitate the electron collection efficiency and hole‐blocking capability from the perovskite layer to the ITO substrate. As a result, the PCE of the modified device reaches 20.55%, much higher than 12.81% of the ITO‐based device, and comparable to state‐of‐the‐art PSCs with an ETL. The efficiency enhancement is originated from the improved *V*_oc_, *J*_sc_, and FF, which is directly related to the decreased series resistance and the increased shunt resistance that implies reduced electron barrier and suppressed nonradiative recombination at the D‐M‐ITO/perovskite interface. Also, the modified device shows a weaker hysteresis and a higher stability under dark in inert gas atmosphere. The key advantages of the present device design are the high conversion efficiency potential with simple device structure and the fact that the whole device production process can be carried out at economical and energy efficient temperatures. Our work highlights the significance of the energy‐level alignment via work function regulation at the interface of ITO/perovskite and sheds light on the design of advanced interfacial materials for simplified PSC devices toward large‐area cost‐effective flexible PSCs applications.

## 4 Experimental Section

##### Materials

Pre‐patterned ITO‐coated glass substrates with a sheet resistance of 15 Ω ▫^−1^ were purchased from Ying Kou You Xuan Trade Co. Ltd., China. The MSAPBS was synthesized according to the previous work.^1^ The perovskite precursors: lead (II) iodide (PbI_2_, 99.99%), lead (II) bromide (PbBr_2_), methylamine hydrobromide (MABr, >98%), and formamidine hydroiodide (FAI, >98%) were purchased from Xi'an Polymer Light Technology Corp, China. DMF (99.8%) and DMSO (99.99%, J&K Reagent) were acquired from Sigma‐Aldrich. Spiro‐MeOTAD (99.9%, Ningbo Borun New Material Technology Co., Ltd.), Li‐bis(trifluoromethanesulfonyl)imide (Li‐TFSI, Acros), 4‐*tert*‐butylpyr‐idine (4‐tBP, Sigma‐Aldrich), acetonitrile, and chlorobenzene were used as received without further purification.

##### Device Fabrication

ITO glass was first cleaned by detergent, acetone, and 2‐propanol in sequence at intervals of 15 min in an ultrasonic bath. After drying with nitrogen flow, ITO glass was treated by UV‐ozone treatment for 15 min before usage. The MSAPBS ML was fabricated by spin‐coating of MSAPBS/methanol solution onto the precleaned ITO glass at room temperature without any further annealing treatment.^1^ A perovskite precursor solution containing 1.15 m PbI_2_, 0.20 m PbBr_2_, 0.20 m MABr, and 1.09 m FAI was prepared by dissolving all of the powders in DMF and DMSO mixed solvents with a volume ratio of 4:1.^2^ Before use, the perovskite precursor was stirred at room temperature for one night. Perovskite absorber layers were deposited based on the anti‐solvent method. Specifically, the perovskite film was realized with a rotation speed of 1000 rpm for 10 s and 4000 rpm for 30 s by spin coating. Then, 300 mL chlorobenzene was quickly dropped 10 s prior to the end of the program. The spiro‐MeOTAD solution was coated at 3000 rpm for 30 s, where 1 mL spiro‐MeOTAD/chlorobenzene (72.3 mg mL^−1^) solution was employed with the addition of 35 µL Li‐TFSI/acetonitrile (260 mg mL^−1^) and 28 µL 4‐tBP. Then, the samples were retained in a desiccator overnight to oxidate the spiro‐MeOTAD. Finally, Au (30 nm) and Ag (70 nm) were sequentially on the spiro‐MeOTAD by thermal evaporation through a shadow mask with a defined active area of 0.04 cm^2^. Electron‐only devices were fabricated with the structure ITO/(MSAPBS)/perovskite/PCBM_60_/Au. PCBM with a concentration of 15 mg mL^−1^ in chloroform was then spin‐coated on top of the perovskite layer at 1200 rpm for 60 s.

##### Characterization

The current density–voltage (*J*–*V*) characteristics of the solar cells were measured under simulated AM1.5 solar illumination using a Newport‐Oriel Sol3A 450 W solar simulator in a nitrogen‐filled glove box. The light intensity was calibrated to give 100 mW cm^−2^ using calibrated silicon reference cell. The *J*–*V* curves were recorded using Keithley 2440 source‐meter. The active area of the solar cells was defined with a metal aperture mask of 0.04 cm^2^. The EQE measurements were carried out using Newport‐Oriel IQE 200, which was calibrated by a standard Si/Ge solar cell. A Dektak 150 Vecco profilometer was used to measure the thickness of different layer.

The SEM diagram and EDS were measured with instrument Verios G4 UC (Thermo Scientific, America). AFM and KPFM images were taken by a Dimension 3100 instrument (Vecco, America) operated. In KPFM, the topography of the film surface was measured in tapping mode, and the tip was held at about 50 nm above the film surface. Contact angle measurement was conducted on a contact angle meter (Data Physics Instruments, OCA20). The XRD measurement was performed at ambient conditions using a Bruker D8 Advanced diffractometer in Bragg‐Brentano geometry and operating with Cu Kα radiation source (*λ* = 1.54 Å). UV‐vis absorption spectrum was taken by a Lambda 950 instrument (Perkin‐Elmer, America). Steady‐state PL and TRPL spectra were acquired using a Horiba Fluorolog fluorimeter (FL3‐111). Samples were excited at 450 nm and the data for each sample were collected under the same conditions. XPS and UPS were conducted on a ultraviolet photoelectron spectrometer (Shimadzu Corporation, Axis Ultra DLD).

## Conflict of Interest

The authors declare no conflict of interest.

## Supporting information

Supporting Information

Supplemental Video 1
