# Supplementary material for: Synergistic Interface Energy Band Alignment Optimization and Defect Passivation toward Efficient and Simple‐Structured Perovskite Solar Cell
Source: Adv Sci (Weinh). 2020 Jan 29;7(6):1902656. doi: 10.1002/advs.201902656 (PMC7080507; doi:10.1002/advs.201902656)
Supplement: Supplementary file 1 — Supporting Information [file ADVS-7-1902656-s001.pdf]

Copyright WILEY-VCH Verlag GmbH & Co. KGaA, 69469 Weinheim, Germany, 2020.

ADVANCED SCIENCE Open Access

Supporting Information

for *Adv. Sci.,* DOI: 10.1002/advs.201902656

Synergistic Interface Energy Band Alignment Optimization and Defect Passivation toward Efficient and Simple-Structured Perovskite Solar Cell

*Like Huang, Danli Zhang, Shixiao Bu, Ruixiang Peng, Qiang Wei, and Ziyi Ge**

Copyright WILEY-VCH Verlag GmbH & Co. KGaA, 69469 Weinheim, Germany, 2020.

ADVANCED SCIENCE
Open Access

Supporting Information

For *Adv. Sci.*, DOI: 10.1002/advs.201902656

Synergistic Interface Energy Band Alignment Optimization and Defect Passivation towards Efficient and Simple-Structured Perovskite Solar Cell

Like Huang,[†] Danli Zhang,[†] Shixiao Bu,[†] Ruixiang Peng,[†] Qiang Wei,[†] Ziyi Ge*,[†,‡]

[†] Ningbo Institute of Materials Technology and Engineering (NIMTE), Chinese Academy of Sciences (CAS), Ningbo, P. R. China

[‡] Center of Materials Science and Optoelectronics Engineering, University of Chinese Academy of Sciences, Beijing, P. R. China

E-mail: geziyi@nimte.ac.cn

Materials and devices characterization as well as interface properties analysis

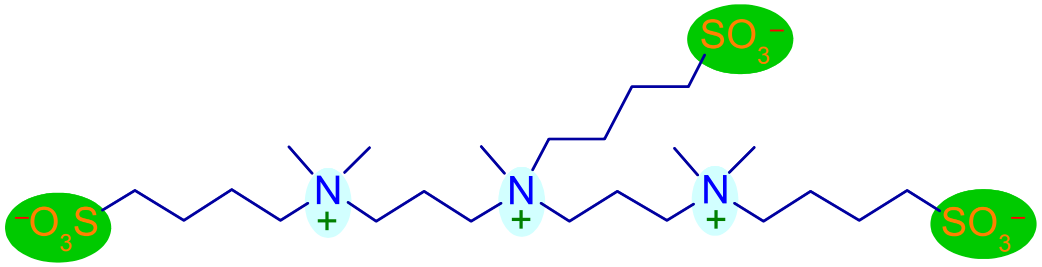

Figure S1 Molecular structure of MSAPBS.

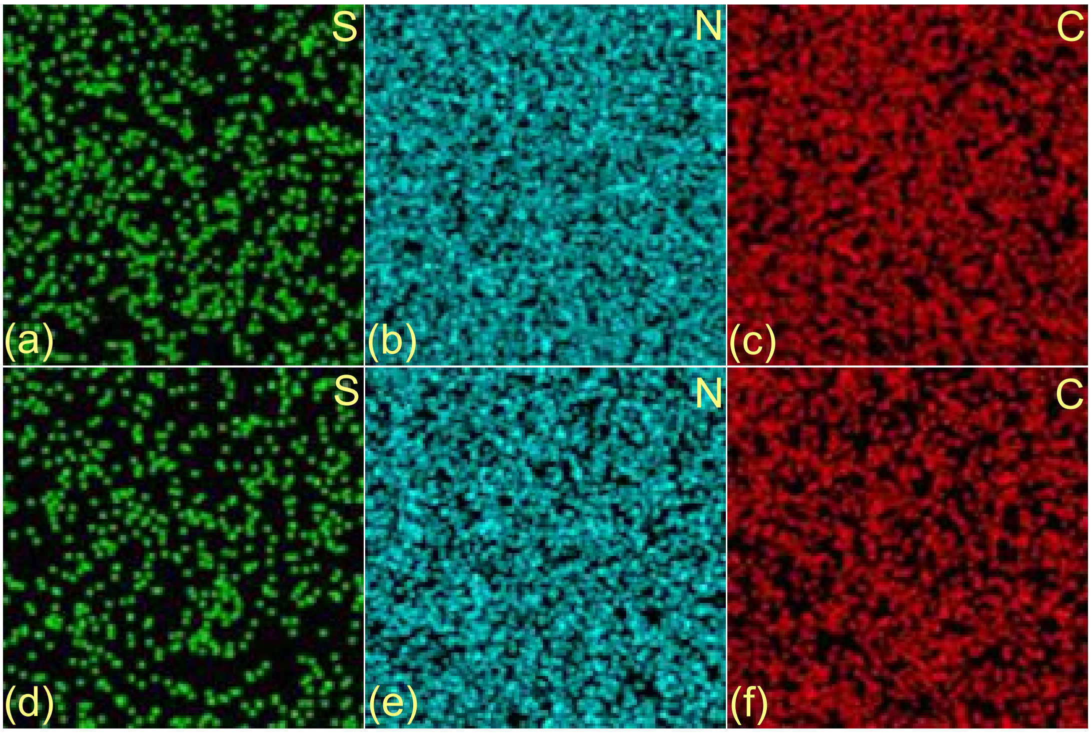

Figure S2 Energy dispersive spectrum (EDS) of MSAPBS films coated ITO before (M-ITO, a-c) and after DMF washing (D-M-ITO, d-f). The scanning area is 2 μm × 2 μm.

From **Figure S2**, the spatial distribution of the characteristic elements (S, N, C) of the MSAPBS film was obviously sparse after being washed with DMF as DMF can wash away part of the MSAPBS film by dissolving the film.

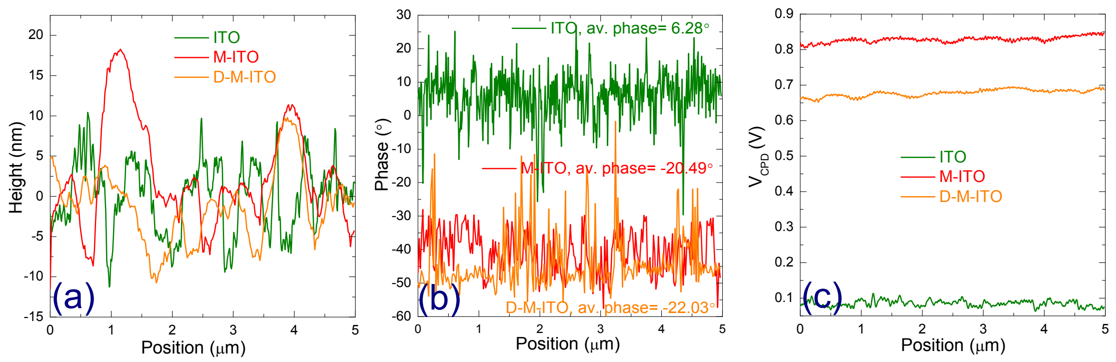

Figure S3 Sectional height change (a), phase change (b) and variations in local CPD across ITO, M-ITO and D-M-ITO surface derived from AFM and KPFM measurement.

The MSAPBS modification improves the surface roughness of ITO due to irregular aggregation of MSAPBS itself thus non-uniform film formation. While the DMF rinse reduces the roughness of the M-ITO surface to the value close to pristine ITO, which can be due to the removal of the surface spike of MSAPBS film (**Figure S3**a).

Also, note that some area of the phase-contrast AFM images of D-M-ITO (**Figure 1**i), which corresponds to the concave area of the MSAPBS film (**Figure 1**f), displays a phase diagram similar to the ITO surface (**Figure 1**g), suggesting that such area can be pin-hole area without MSAPBS coverage. This can also be the reason why the SEM image of D-M-ITO looks slightly white than the that of M-ITO.

Phase-contrast imaging in the tapping mode AFM is a powerful method in surface characterization, which can provide fine details about rough surfaces of their microstructure and micro-area composition that are normally obscured in topographic imaging. Certain surface properties including friction, adhesion and viscoelasticity, affect the magnitudes of the phases that are produced by a surface. Phase contrast also arises from compositional variations of the surface as well as the topographical variations caused by changes in adhesion between the tip and the specimen surface[3]. From **Figure S3**b, the average phase of pristine ITO is 6.28°, MSAPBS modification decreases the average phase to -20.49°. While, the average phase of the D-M-ITO (-22.03°) is close to that of the M-ITO, but with larger oscillation amplitude compared to M-ITO. In some area, the phase of D-M-ITO even approaches the value of the

pristine ITO (**Figure S3**b), suggesting these area can be pin-holes or deep cave with very little MSAPBS covered as mentioned above.

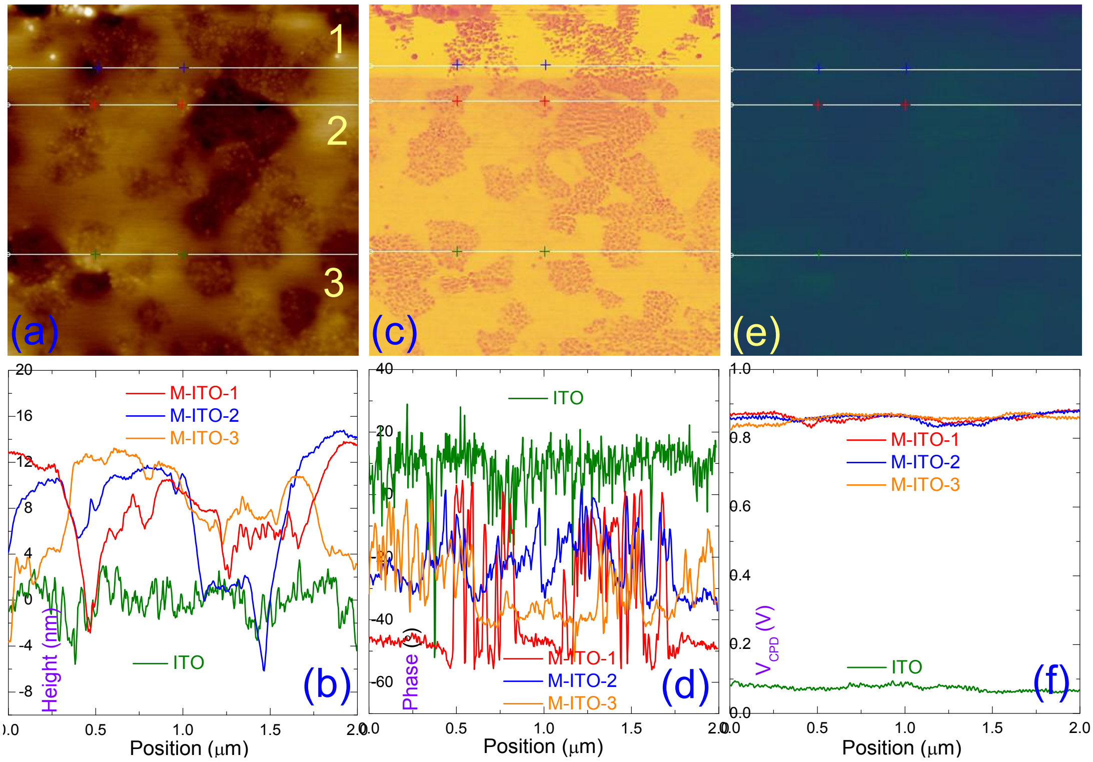

Figure S4 Topographic AFM images (a), phase-contrast AFM images (c) and CPD (e) of M-ITO. The area of all the images is 2 μm × 2 μm. Sectional change of height (b), phase (d) and CPD (f). Note that in order to facilitate the comparison and discussion, the height in (b) was added by 10 nm (which is the thickness of the MSAPBS film).

In order to further confirm the uniform of the work function of the ITO surface by MSAPBS modification, **Figure S4** further gives the high resolution topographic AFM images (a), phase-contrast AFM images (c) and CPD (e) of M-ITO. The apparent thickness and composition inhomogeneity (**Figure S4**a, c) seem to have little effect on the surface potential of the final film (**Figure S4**e). In **Figure S4**b, d, f, we give the height, phase and potential change along three representative lines. The sectional height along the three lines were added by 10 nm. Thus, one can find that there exist pin-hole area in the MSAPBS film as the height line of the three overlap with that of ITO (**Figure S4**b). From **Figure S4**b, the change of phase and height shows the

opposite trend, which is consistent with the fact that the change of surface hardness and viscosity is caused by the change of film thickness. All the potential change along the three lines are small, in spite of the larger morphological and phase change of M-ITO as mentioned above. Also, the potential along the three lines have little difference on value. These results suggest that the MSAPBS film coverage with the presently discussed thickness hardly affects the homogeneity of M-ITO work function. The specific underlying mechanism is not entirely clear presently, further deep research is under way and will be published elsewhere.

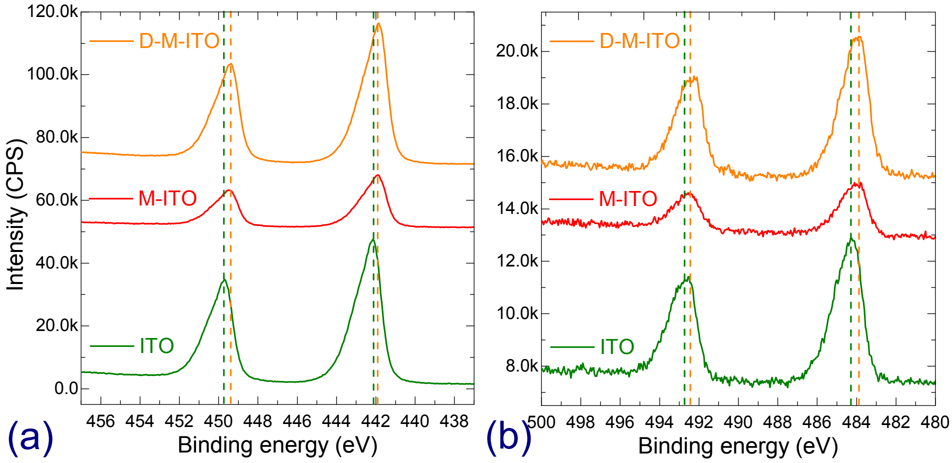

Figure S5 XPS measurement of the pristine ITO, M-ITO and D-M-ITO: (a) In 3d , (b) Sn 3d.

Figure S5 gives the In 3d and Sn 3d core levels XPS, from which we can see that the main peaks intensity change and shift have the same trend for In 3d and Sn 3d core levels. In terms of intensity change, the modification screens part of the element information of the underlying ITO. Therefore, both of the intensity of In 3d and Sn 3d core levels decreases for M-ITO. As XPS is a surface-sensitive analytical technique and it can only give the information of the sample surface on several nanometer scale, the existence of the In 3d and Sn 3d peaks can be due to the incomplete coverage of MSAPBS on ITO surface as suggested by SEM and AFM images (**Figure 1**, **Figure S2** and **Figure S4**). Further DMF washing removes some MSAPBS and further decease its coverage and thickness and the signal of the underlying ITO increase then.

In terms of peak position shift, both peaks of In 3d and Sn 3d core levels moves toward lower binding energy after modification, indicating a chemical interaction of MSAPBS molecules with ITO, possibly due to the formation of Sn-S and In-S bonds[4]. Interestingly, this chemical shift for In 3d and Sn 3d core levels of D-M-ITO maintain even after DMF washing, which suggests the strong chemical adsorption of MSAPBS molecules on ITO surface possibly due to the Sn-S and In-S bonds as mentioned above. In previous work, Sn-N bond was suggested as the origin of interface dipole moment[5], however in our work here the modifier adopted is a polar molecule. In this case it is impossible to exclude this chemical bond (Sn-S and In-S bonds) as one of the origins of the interface dipole. However, it is certain that the interface dipole originates mainly from the dipole of the MSAPBS molecule itself as suggested by the fact that the work function of the modified ITO depends on the thickness of the modifier, which will be discussed in detail elsewhere.

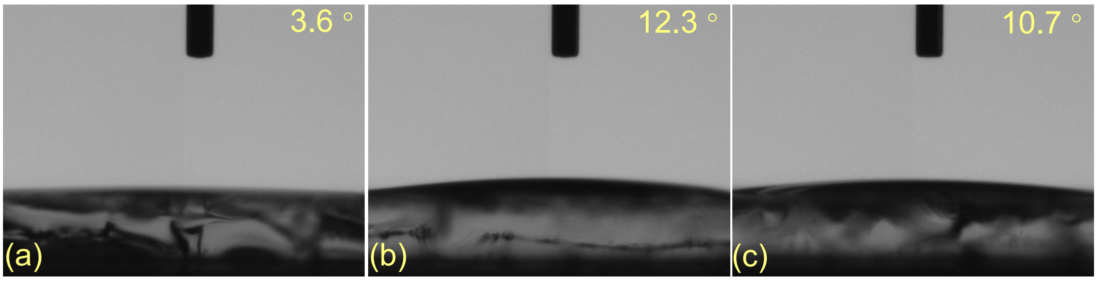

Figure S6 Contact angle measurement. (a) ITO after UV-O_3 treatment, (b) MSAPBS coated ITO (M-ITO) and (c) MSAPBS coated ITO with DMF washing (D-M-ITO).

Although the MSAPBS film coverage hardly affects the homogeneity of M-ITO work function thus the device performance. While, our previous work have shown that perovskite coverage has a significant impact on the device performance of ETL-free PSCs[6, 7]. From **Figure S7**, both of the perovskite films deposited on ITO and D-M-ITO are densely packed and exhibit full coverage without pinholes and grain boundary crack.

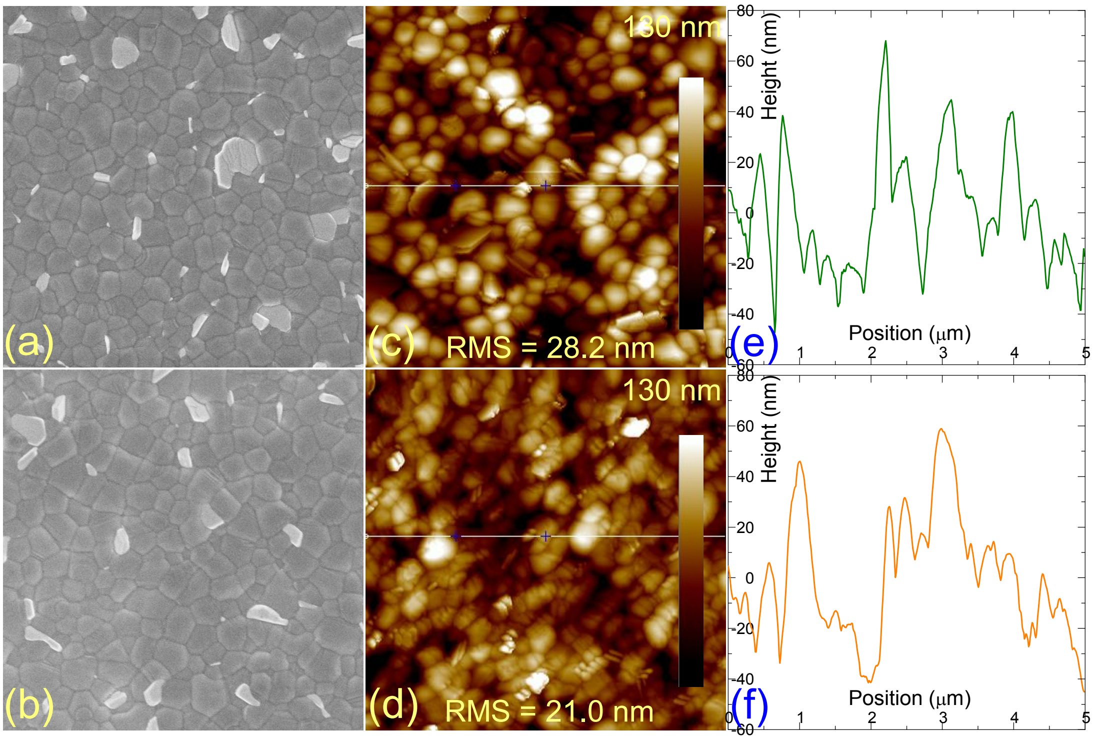

Figure S7 SEM images of perovskite film deposited on (a) ITO and (b) D-M-ITO. Topographic AFM images of perovskite film deposited on (c) ITO and (d) D-M-ITO. Sectional height change of perovskite film deposited on (e) ITO and (f) D-M-ITO. The scanning area of all the images is 5 μm × 5 μm.

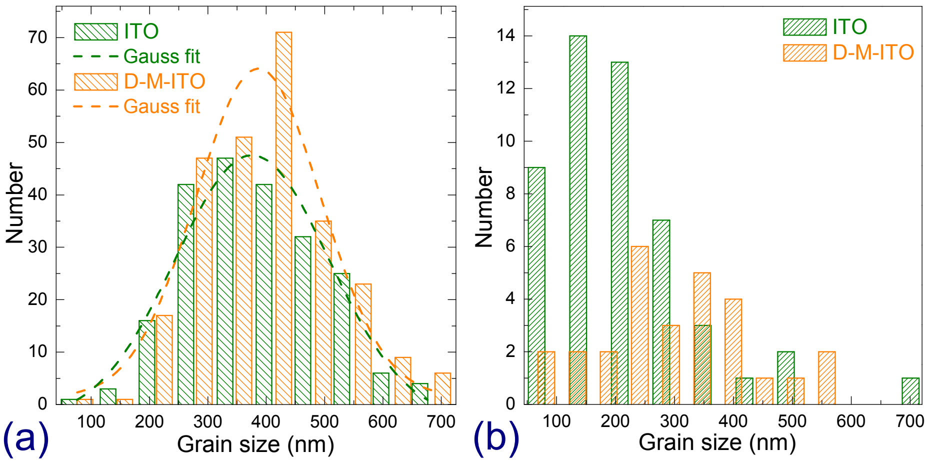

Figure S8 Statistical distribution of grain sizes of perovskite films (a) and remnant PbI_2 phase (b).

From **Figure S8**a, the perovskite film deposited on the pristine ITO has a maximum and minimum grain size of 688.37 and 8.36 nm, respectively. While the perovskite

film deposited on D-M-ITO has a maximum and minimum grain size of 709.47 and 42.63 nm, respectively. This contributes to an enlarged average grain size from 363.90 to 385.59 nm, which is well consistent with the results of XRD (**Figure S9**) and SEM (**Figure 3**a, b and **Figure 7**a, b), and can be due to the fact that the slightly hydrophobic modification layer (relative to ITO itself) reduces the nucleation rate and density of perovskite precursor during solvent evaporation. The interaction between modified layer and perovskite precursor also inhibits the formation of excessive residual PbI_2 (the 'white phase' in **Figure 3**a, b and **Figure 7**a, b) as a second phase, in spite of the previous report that excess PbI_2 could passives the defects and reduce recombination in the perovskite film[8]. From **Figure S8**b, the perovskite film deposited on the pristine ITO contains PbI_2 nanocrystal with a maximum and minimum grain size of 749.80 and 45.17 nm, respectively. While the perovskite film deposited on D-M-ITO contains PbI_2 nanocrystal with a maximum and minimum grain size of 570.03 and 42.09 nm, respectively. This is well consistent with the results of XRD in which a much sharper diffraction peak that corresponds to PbI_2 was observed for the perovskite film deposited on D-M-ITO (**Figure S9**a).

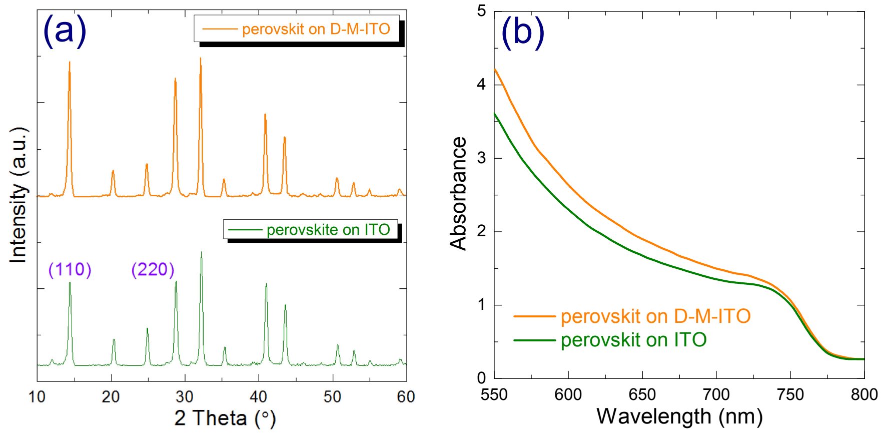

Figure S9 XRD (a) and UV-vis absorbance spectrum (b) of the perovskite films deposited on the pristine ITO and D-M-ITO.

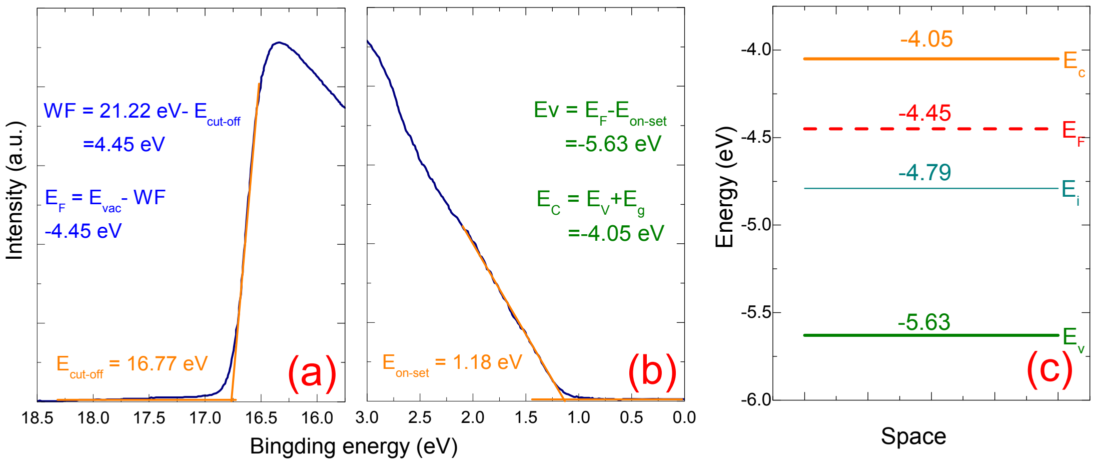

Figure S10 UPS spectra of perovskite film in the secondary-cutoff region (a) and in the valance band region (b). The energy level position derived from the UPS measurement (c).

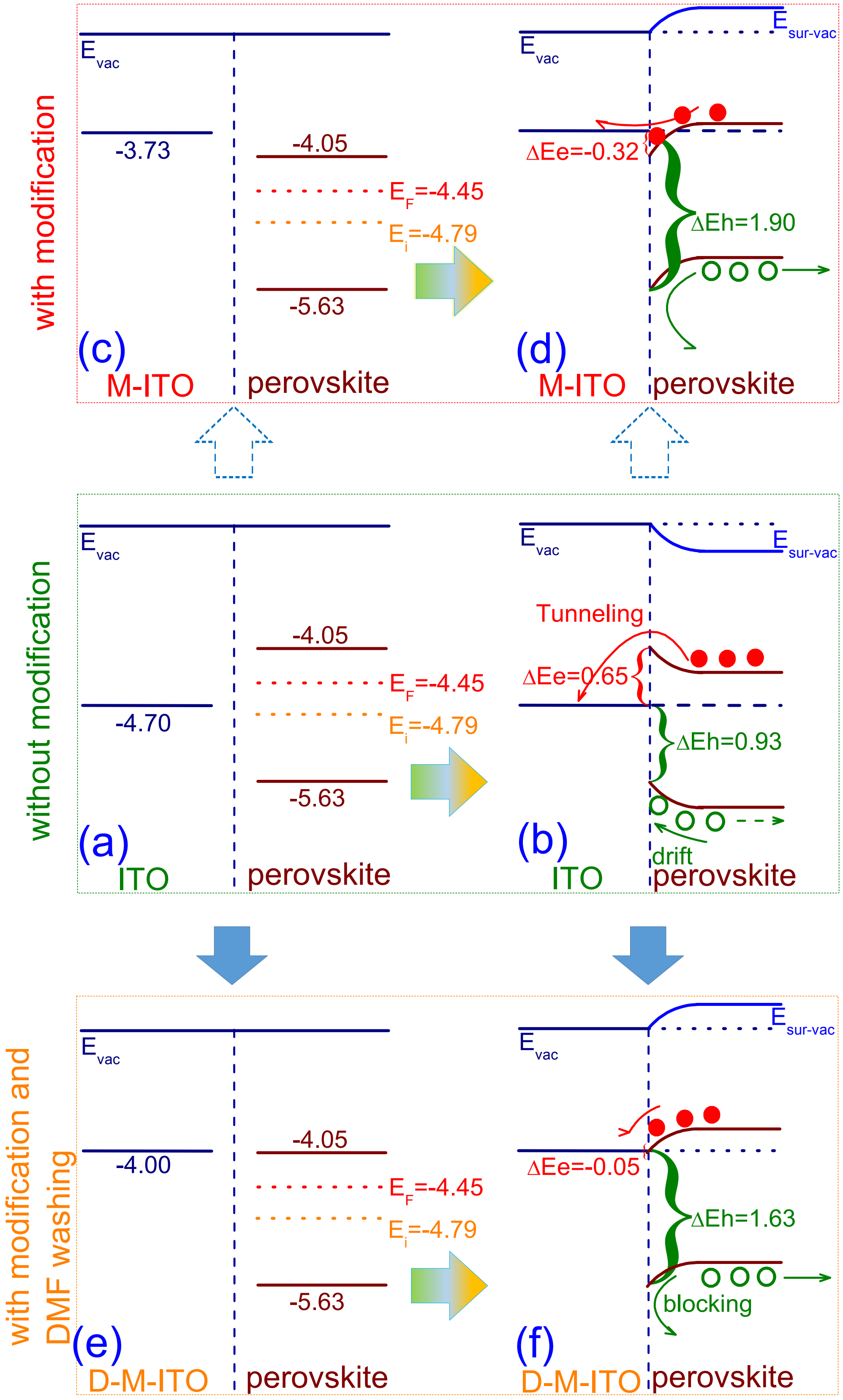

Figure S11 Energy levels alignment of ITO/perovskite interface before and after contact: (a, b) without modification, (c, d) with modification (theoretically) and (e, f) with modification and DMF washing (actual situation).

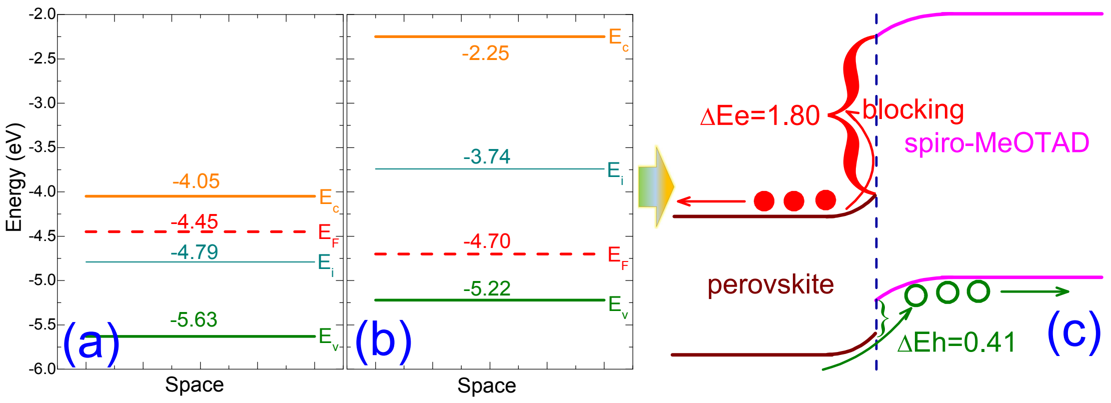

Figure S12 Energy levels of isolated $(FAPbI_3)_{0.85}(MAPbBr_3)_{0.15}$ perovskite (a) and spiro-MeOTAD HTL (b) before contact. Energy levels alignment of the n-type perovskite and p-type spiro-MeOTAD HTL after contact (c).

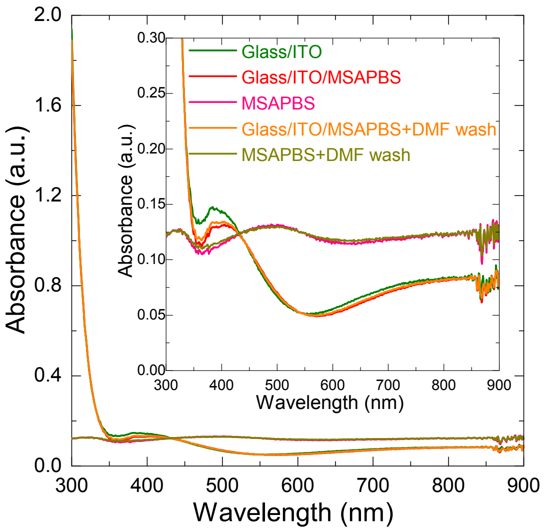

Figure S13 Absorbance spectrum of the pristine ITO, M-ITO and D-M-ITO.

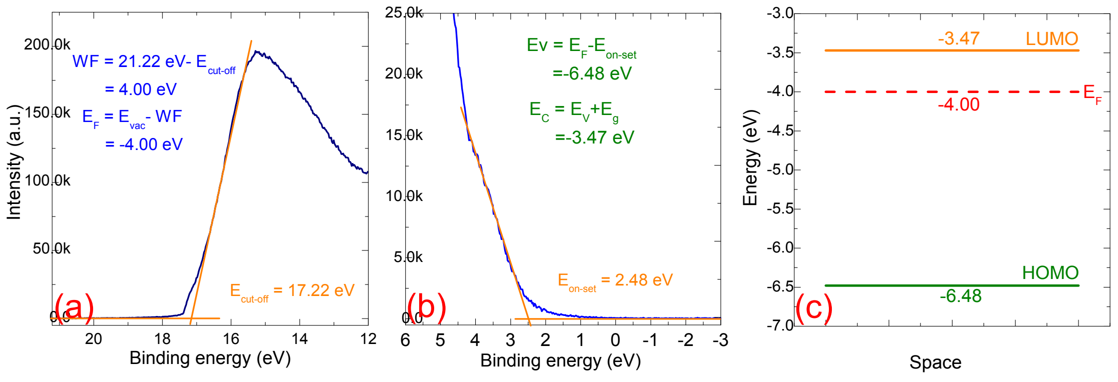

Figure S14 UPS spectra of MSAPBS film in the secondary-cutoff region (a) and in the valance band region (b). The energy level position derived from the UPS

measurement (c).

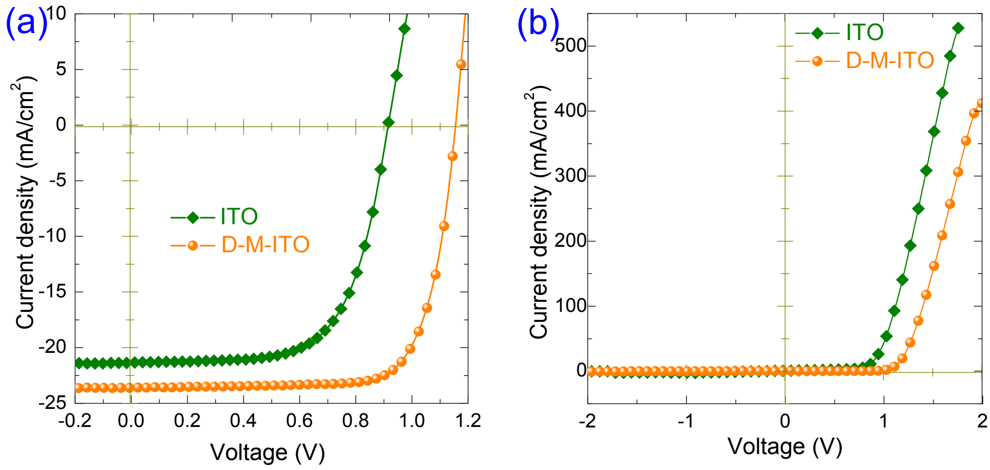

Figure S15 *J-V* curves (reverse scan) of ETL-free PSCs with and without MSAPBS modification under AM1.5 solar illumination (a) and dark (b).

To quantify the degree of *J-V* hysteresis, the following hysteresis factor (HF) defined in previous work (Equation S1)[9] is adopted.

$$HF = \frac{PCE_{reverse} - PCE_{forward}}{PCE_{reverse}} \quad (S1)$$

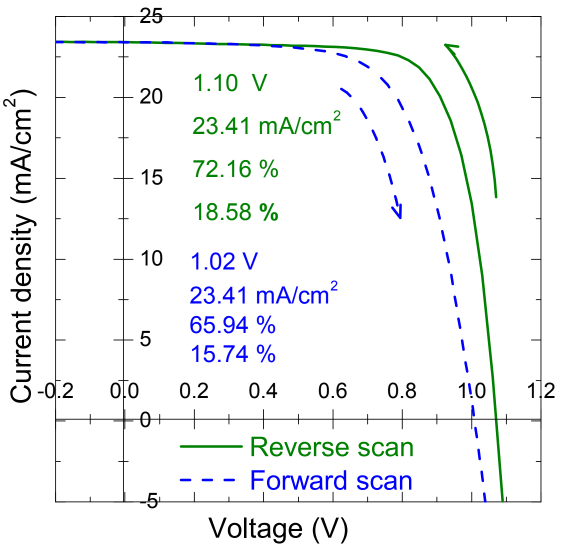

Figure S16 *J-V* curves of PSCs based on nanocrystalline SnO_2 electron transport layer. According to equation (S1), the hysteresis factor for such a device is 0.15, which is a

common value for planar heterojunction PSC as reported previously.

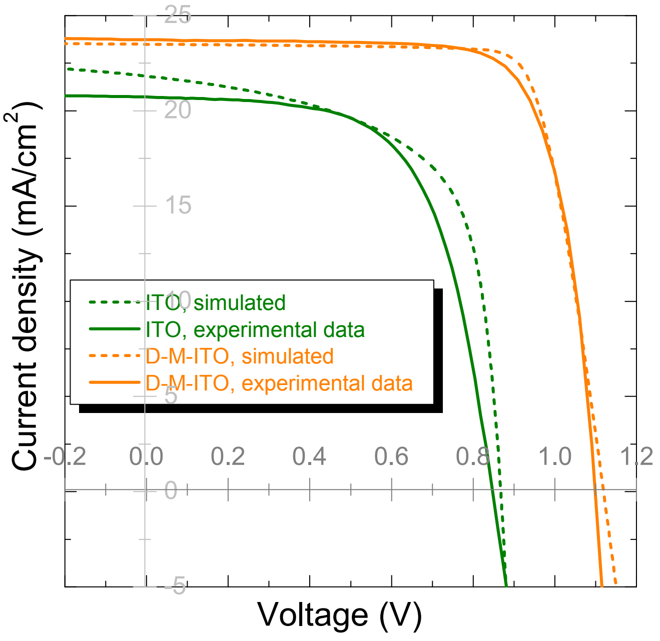

Figure S17 *J-V* curves of ETL-free PSCs with and without MSAPBS modification derived from real experiments (solid line) and device simulations (dotted line).

Device simulation based on 1-dimensional simulation program SCAPS was also conducted (Solar Cell Capacitance Simulator)[10] to further reveal the effect of the ITO work function on the device performance. As shown in **Figure S17**, *J-V* curves of ETL-free PSCs with and without MSAPBS modification derived from experiments (solid line) and device simulations (dotted line) are presented, a small difference can be observed between experimental dates and simulation results (**Table S1**).

Table S1. Photovoltaic parameters of ETL-free PSCs with and without MSAPBS modification derived experiments and simulations.

Device	Date source	Voc [V]	Jsc [mA/cm^2]	FF [%]	PCE [%]
ITO	Experiment	0.85	20.71	62.80	11.05
	Simulation	0.87	21.82	63.08	11.97
D-M-ITO	Experiment	1.10	23.74	73.94	19.31
	Simulation	1.12	23.50	77.87	20.13

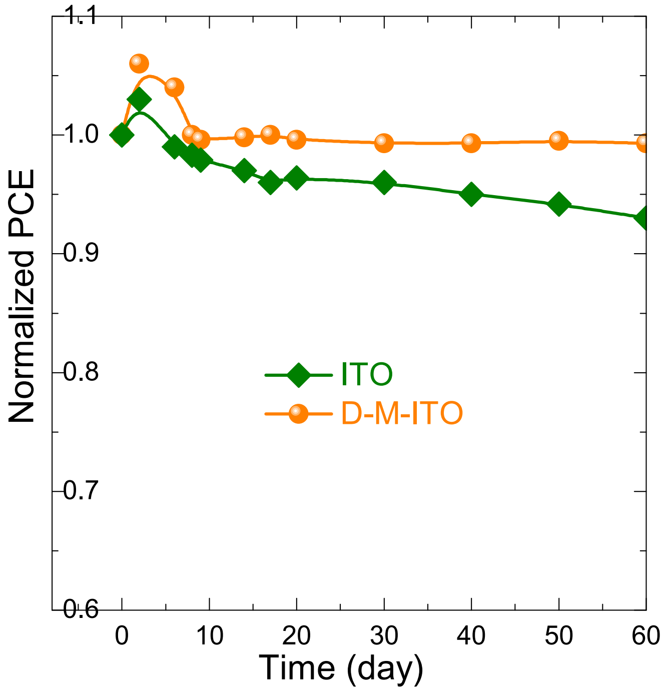

Figure S18 Stability comparison of ITO and D-M-ITO based ETL-free PSCs stored in dark in glove-box filled with N_2 (Oxygen and water content less than 0.1 ppm).

Demonstration of the universality of the present electrode design strategy

In this part, we will show the universal applicability and advantages of the current electrode design with other three kind of TCO electrode/perovskite combination.

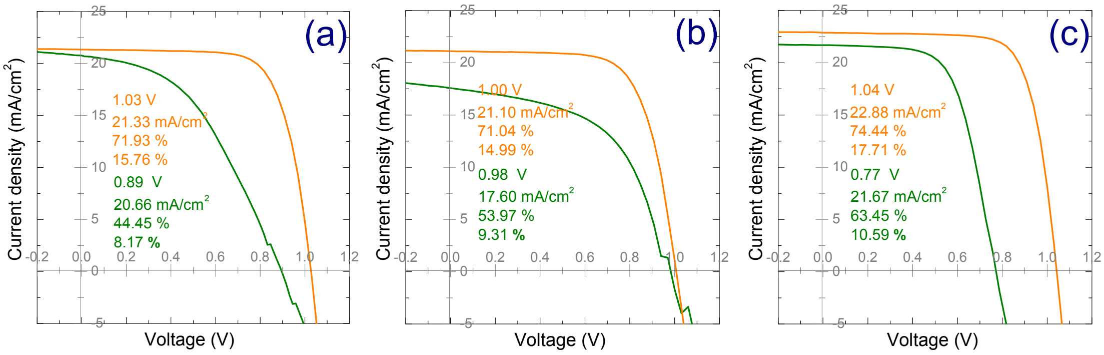

Figure S19 *J-V* curves of ETL-free PSCs with and without MSAPBS modification: (a) ITO/$MAPbI_3$, (b) FTO/$MAPbI_3$, and (c) FTO/$(FAPbI_3)_{0.85}(MAPbBr_3)_{0.15}$. All curves are obtained from reverse scan under AM1.5G (100 mW/cm^2).

Table S2. Photovoltaic parameters of different ETL-free PSCs with and without MSAPBS modification.

Electrode	Perovskite	*V*oc [V]	*J*sc [mA/cm^2]	FF [%]	PCE [%]
ITO	$MAPbI_3$	0.89	20.66	44.45	8.17
	$(FAPbI_3)_{0.85}(MAPbBr_3)_{0.15}$	0.92	21.34	65.27	12.81
D-M-ITO	$MAPbI_3$	1.03	21.33	71.93	15.76
	$(FAPbI_3)_{0.85}(MAPbBr_3)_{0.15}$	1.15	23.62	75.67	20.55
FTO	$MAPbI_3$	0.98	17.60	53.97	9.31
	$(FAPbI_3)_{0.85}(MAPbBr_3)_{0.15}$	0.77	21.67	63.45	10.59
D-M-FTO	$MAPbI_3$	1.00	21.10	71.04	14.99
	$(FAPbI_3)_{0.85}(MAPbBr_3)_{0.15}$	1.04	22.88	74.44	17.71

From **Figure S19** and **Table S2**, MSAPBS modification can significantly improve the device performance of ETL-free PSCs, regardless of the specific combination of TCO electrode and perovskite adopted, which fully confirms the universality of the current strategy of electrode design.

Cost analysis of material and device

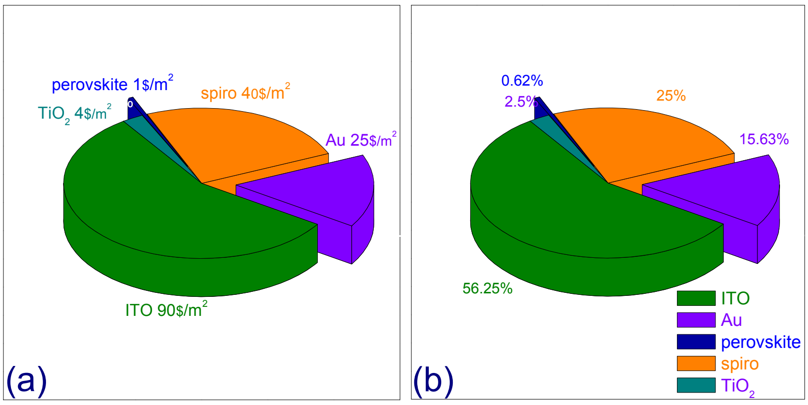

Figure S20 Pie chart displaying the estimated cost (a) and cost ratio (b) of the different layers in traditional planar PSCs with TiO_2 as ETL.

Cost analysis for PSC (**Figure S20**) is roughly conducted according to reported works[11]. From the figure, the cost of ETL only accounts for 2% of the total cost of traditional planar devices, although the modification materials we use are much cheaper than TiO_2 due to its simple synthesis process and extremely limited dosage, there is limited room for cost reduction. The advantages of the current device design are mainly embodied in the following aspects:1. Its superiority over mesoporous PSCs with much more complex structure design and device process hence much higher cost; 2. Its great potential in flexible applications (which will be demonstrated elsewhere) due to its much simpler device structure hence simpler fabrication process and lower processing temperature; 3. Its readily recyclability with much simple device structure. In particular, from **Figure S20** the ITO front electrode, hole transporting material and Au back electrode are three significant cost factors. With the much limited abundance of indium and gold element, recycling of these material can be significantly meaningful. Here we will briefly verify the recyclability of such devices.

Demonstration of the recyclability of the present device

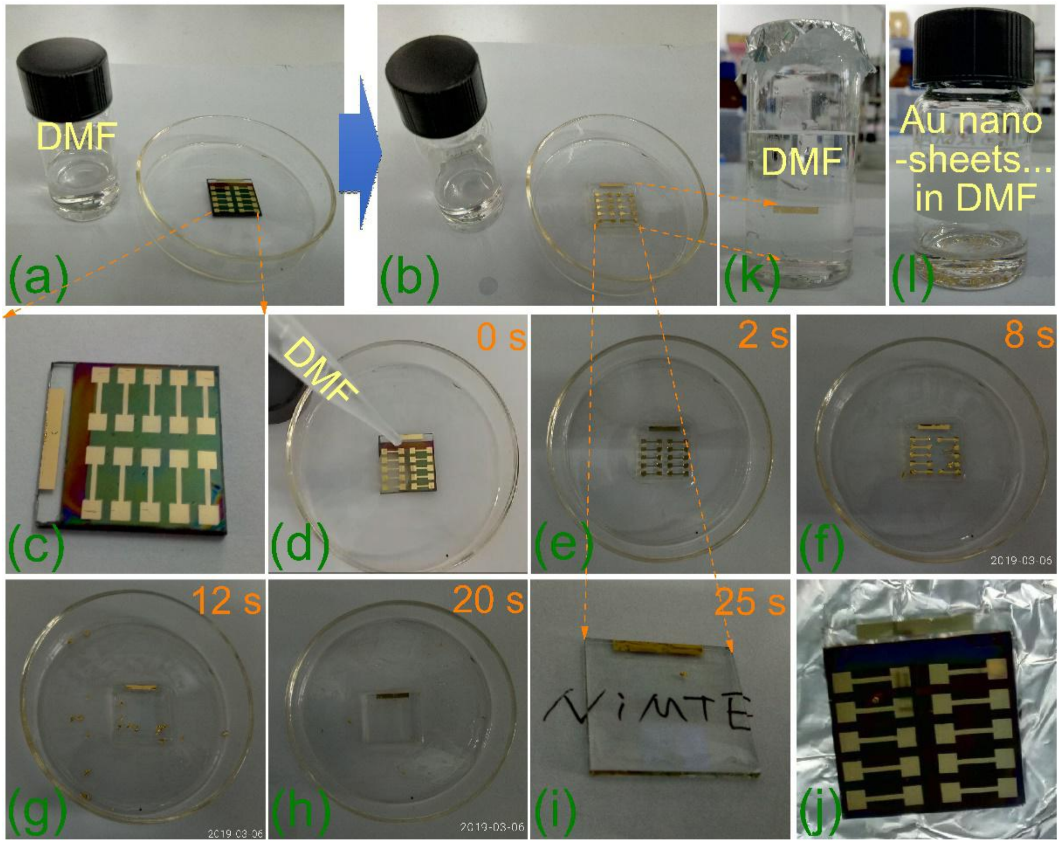

Figure S21 The process flow to fabricate ETL-free PSCs by recycling the ITO/glass substrates from the degraded devices based on D-M-ITO. Degraded device before (a) and after (b) DMF washing. Time lapse sequence of washing and soaking the PSCs in DMF (d-i, k). Optical photographs of the original device (c) and new device based on the reused M-ITO substrates (j). Gold nanoflakes that are dispersed in the DMF solution (l).

Figure S21 gives the process flow to fabricate efficient ETL-free PSCs by recycling the glass/ITO substrate from degraded devices. Due to its strong solubility, DMF can easily dissolve spiro-MeOTAD and perovskite film, while the insoluble gold electrode film will float on the surface of the DMF solution (**Video 1**). The suspended gold films can be easily filtered from the DMF solution for reusing. The element Pb in the blend solution can be converted to PbI_2 by physical and chemical methods and can be participated in the new round of device fabrication, thus avoiding

the environmental pollution caused by possible lead out flowing. By proper processing including DMF washing (**Figure S21** d-i), DMF soaking (**Figure S21** k), ultrasonic cleaning and UV-O_3 treatment, the recycled substrate is still very clean and transparent and can be used for fresh device fabrication.

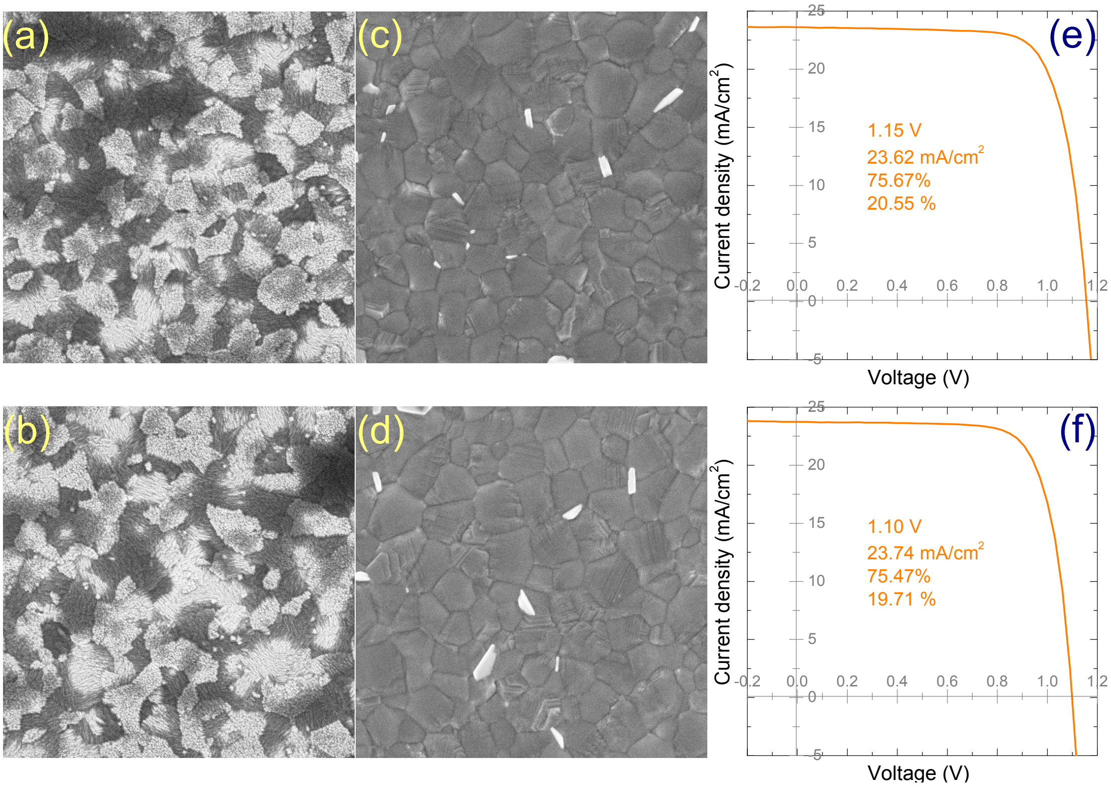

Figure S22 SEM images of the original (a) and the reused (b) M-ITO substrates, SEM images of the perovskite films deposited on the original (c) and the reused (d) M-ITO substrates. The area is 3 μm × 3 μm. *J-V* curves of ETL-free PSCs based on the original (e) and the reused (f) M-ITO substrates. All curves are obtained from reverse scan under AM1.5G (100 mW/cm^2).

Reference

[1] X. Ouyang, R. Peng, L. Ai, X. Zhang, Z. Ge, *Nature Photonics* **2015**, 9, 520.

[2] D. Bi, W. Tress, M.I. Dar, P. Gao, J. Luo, C. Renevier, K. Schenk, A. Abate, F. Giordano, J.P.C. Baena, *Science Advances* **2016**, 2, e1501170.

[3] G.K.H. Pang, K.Z. Baba-Kishi, A. Patel, *Ultramicroscopy* **2000**, 81, 35.

[4] Y. Zheng, T. Zhou, C. Zhang, J. Mao, H. Liu, Z. Guo, *Angew Chem Int Ed Engl* **2016**, 55, 3408.

[5] W. Kong, W. Li, C. Liu, H. Liu, J. Miao, W. Wang, S. Chen, M. Hu, D. Li, A. Amini, S. Yang, J. Wang, B. Xu, C. Cheng, *ACS Nano* **2019**, 13, 1625.

[6] L. Huang, J. Xu, X. Sun, Y. Du, H. Cai, J. Ni, J. Li, Z. Hu, J. Zhang, *ACS Applied Materials & Interfaces* **2016**, 8, 9811.

[7] L. Huang, S. Bu, D. Zhang, R. Peng, Q. Wei, Z. Ge, J. Zhang, *Solar RRL* **2019**, 3, 1800274.

[8] Q. Jiang, Z. Chu, P. Wang, X. Yang, H. Liu, Y. Wang, Z. Yin, J. Wu, X. Zhang, J. You, *Advanced Materials* **2017**, 29, 1703852.

[9] L. Zhen, J. Tinkham, P. Schulz, M. Yang, Z. Kai, *Advanced Energy Materials* **2016**, 6, 1601451.

[10] S. Soedergren, A. Hagfeldt, J. Olsson, S.E. Lindquist, *Journal of Physical Chemistry* **1994**, 98, 5552.

[11] A. Binek, M.L. Petrus, N. Huber, H. Bristow, Y. Hu, T. Bein, P. Docampo, *ACS Applied Materials & Interfaces* **2016**, 8, 12881.
